# Cyber-Physical System Interface for Implantable Esophageal Prosthesis

**DOI:** 10.3390/s25144469

**Published:** 2025-07-18

**Authors:** Ana Magdalena Anghel, Teodora Mîndra

**Affiliations:** Faculty of Automatic Control and Computers, National University of Science and Technology POLITEHNICA Bucharest, 313 Splaiul Independenței, 060042 Bucharest, Romania; mindrateodora@gmail.com

**Keywords:** Cyber-Physical System Interface, esophageal prosthesis, implants

## Abstract

This article presents a Cyber-Physical System Interface (CPSI) for a patented implantable esophageal prosthesis. Designed for in vivo use, the CPSI has been implemented in a MATLAB (version R2021b) simulation environment integrated with real-time data from sensors relevant for monitoring the prosthesis’s physical positioning and environmental interactions, aggregated through an Arduino external system. This setup enables the modeling and analysis of system behaviors in a controlled setting. The paper discusses the sensors, hardware and software components supporting a wide range of applications, and the method chosen for sensor-to-display flow. The case study demonstrates two monitoring system applications: one analyzes the influence of variations in the prosthesis geometry, while the other evaluates the tissue response to the implant. The proposed framework and implementation are highly relevant for a wide range of in vivo implants and related systems.

## 1. Introduction

Recent research indicates that gastroesophageal cancer is the fourth most common gastrointestinal cancer after colorectal, pancreatic, and hepatobiliary cancer and has the third highest mortality rate [1]. At the same time, technological advances [2] have brought numerous results in increasing the chances of survival of patients with various diseases, making people aware of the diagnosis with positivity, and being more focused on treatment.

The storage of patient data and their monitoring both in real time and as a medical history, have enabled more complex analysis that helps the doctor to predict early a possible relapse. This fact has increased the need to develop data collection and acquisition systems, either in a centralized or decentralized way, depending on their architecture and the way they manage the data.

This article addresses the challenge of integrating a real-time, versatile Cyber-Physical System Interface (CPSI) for in vivo monitoring of implantable medical devices. Current systems often lack modularity, adaptability to various sensor types, or the ability to visualize and analyze data in real time in a simulation environment. Our proposed framework combines a widely available controller (Arduino) with MATLAB-based visualization, creating a modular system that can be adapted to different application domains, such as medicine, energy, or pharmaceuticals.

For instance, in energy monitoring, the same system architecture (based on sensors and CPSI) could be adapted to track environmental conditions around energy infrastructure (e.g., vibration sensors for pipeline monitoring, temperature sensors for overheating detection, or tilt sensors for stability control of solar panels).

In the pharmaceutical field, the CPSI can be used to monitor environmental variables (temperature, humidity, and vibration) during storage or transport of sensitive materials, ensuring compliance with quality standards.

This cross-domain applicability stems from the generic design of the system, which supports plug-and-play sensor integration, real-time communication with external analysis platforms (such as MATLAB), and flexible data processing pipelines—all essential features of a modern cyber-physical system.

The main contributions of this paper are as follows:

- The development of modular CPSI architecture for real-time data acquisition and analysis, suitable for in vivo applications.
- A practical implementation using a simulation environment (MATLAB) and an Arduino-based sensor system.
- A dual case study demonstrating the system’s utility: (i) analyzing the influence of prosthesis geometry variations and (ii) evaluating tissue response to an implant.

Compared to the usual approaches, which typically focus either on the hardware or software components in isolation, our approach demonstrates salability and adaptability of the system in a medical use case, with potential for cross-domain application.

Despite significant technological and medical advances in the field of esophageal cancer, current research remains heavily focused on areas such as imaging, diagnosis, and surgical intervention. These domains benefit from mature methodologies and are supported by well-established clinical protocols and regulatory frameworks. In contrast, real-time, personalized monitoring using portable or implantable devices integrated into esophageal prostheses is still an underdeveloped segment. This is due to anatomical challenges (e.g., the deep and variable positioning of the esophagus), inter-patient variability, and the absence of standardized safety regulations for such embedded systems. Consequently, CPSI, which offers a promising bridge between sensor networks and remote clinical platforms, has not yet been sufficiently investigated in this context. This work aims to highlight this gap and propose a versatile CPSI-based architecture capable of supporting future development of personalized, real-time monitoring tools for esophageal cancer management.

## 2. Related Work

Technology has brought significant advances in the treatment of esophageal cancer, influencing all stages of the therapeutic process, from diagnosis to treatment and post-treatment monitoring, which has led to a significant improvement in the prognosis of patients. The following section provides an overview of the current state of the art in relation to both diagnosis and advanced treatments and methods for monitoring and increasing the quality of life of patients.

### 2.1. Early Diagnosis

Advanced techniques in esophageal surgery are presented in [3], where Jung K, Haug RM, and Wang AY highlight both the clinical applications of these procedures and recent advances. Goda K and colleagues reported in [4] a study on the application of advanced endoscopy with a special focus on non-erosive reflux disease and eosinophilic esophagitis. In this way, the authors highlight recent advances in benign esophageal diseases, along with diagnostic and therapeutic advances in various conditions. Paper [5] explored advanced imaging and artificial intelligence. Authors support the development of innovative technologies by direct contributions to the current state of the art in the diagnosis and management of Barrett’s esophagus, as well as pinpointing future directions for development in this field. In the article [6], recent advances in the diagnosis and management of esophageal cancer are reviewed. Modern screening and imaging techniques, advances in the analysis of emerging biomarkers, and advanced staging strategies are addressed by the authors. The research also includes new hypotheses for the widespread use of multimodal treatments, including neoadjuvant therapy, minimally invasive surgery, and immunotherapy. All these new multimodal treatments discussed in the paper are addressed simultaneously, highlighting their impact on the prognosis of patients. This led the authors to develop a procedural way of working, thus, providing an updated perspective on clinical guidelines and technological innovations that improve early detection and therapeutic options for this pathology. Wykypiel H and collaborators explore in [7] the clinical implementation of minimally invasive esophagectomy. The article analyzes the advantages, challenges, and results of this technique compared to traditional surgical interventions. The authors propose a detailed set of criteria for the new techniques. They detail aspects such as patient selection, surgical techniques used, perioperative management, and postoperative recovery, highlighting the benefits of this approach, including reduced complications and improved prognosis for patients with esophageal diseases.

The study by Bjelovic M et al. [8] presents a safe approach for the transition from open esophagectomy to minimally invasive total esophagectomy in the treatment of esophageal cancer, using a process management methodology. The authors of this study analyze the main steps for implementing this change and the advantages brought to patients, such as reduced complications and recovery time, but also the challenges encountered in adopting this advanced technique. Although the cited works cover innovative diagnostic techniques and minimally invasive surgeries, they do not address the integration of Cyber-Physical Systems (CPS) for real-time monitoring or post-surgical evaluation. These studies focus on engineering solutions for implantable systems. Our work complements this body of research by introducing a CPS-based interface specifically for evaluating implant performance and physiological response in vivo.

### 2.2. Monitoring and Enhancing Life Quality

Recent studies such as [9] analyze methods for measuring and improving the quality of esophageal care and swallowing disorders, which highlight the main essential performance indicators, treatment optimization strategies are shown, and the role of advanced technologies in diagnosis and management is indicated. This study highlights the importance of multidisciplinary approaches in improving the outcomes of patients with esophageal diseases in terms of their clinical monitoring and personalization of treatments. Also, in the paper [10]. Qiu LH and collaborators investigate quality of life indicators and their impact on the prognosis of patients with esophageal cancer undergoing curative resection. The novelty of the study highlights the importance of continuous clinical monitoring and personalized interventions for each patient to improve long-term outcomes. In this regard, factors influencing the postoperative recovery of patients are analyzed, persistent symptoms and nutritional status are addressed and esophageal function through specific analyses are described.

A comprehensive review of devices used to test esophageal function is provided by Pannala R et al. and is presented in [11]. The study explores available technologies, such as high-resolution manometry, impedance-pH-metry, and motility assessment tests, highlighting the advantages of each method in diagnosing esophageal disorders. Topics such as recent innovations and future directions for improving the accuracy and clinical applicability of these devices are addressed to highlight the direction of modern medicine and its current branches.

Also, in line with modern patient monitoring technologies, the study [12] discusses monitoring technologies, including the use of pH-meter catheters and wireless recording systems, bringing the reader closer to the topics and the importance of accurate assessment of esophageal acidity for successful treatments.

The focus of these studies is on clinical outcome measurement and diagnostic device performance, but they do not explore an integrated CPS platform capable of simulating and visualizing sensor-based data interactions in real time. Our system bridges this gap by offering a flexible, modular solution that allows real-time simulation and communication between sensors and processing platforms—critical for long-term implant evaluation and monitoring.

Previous studies have led the authors to continue their previous studies in this paper. The authors presented in [13] a hybrid co-simulation framework for applications based on cyber-physical systems. The article details the use of co-simulation for modeling communication systems, time series analysis, and the development of predictive models for microgrids and prosthetics. The authors emphasize the integration of real-time communication processes, security analysis, and human interaction in cyber-physical systems. Further, the paper [14] details the integration of the cyber-physical perception interface within co-simulation, underlining the importance of this approach for the development of accurate and interactive models between physical and cyber systems. The study emphasizes the applicability of this interface in various fields, such as real-time monitoring, automation, and control systems.

## 3. Use Case

The project team started from a prosthesis that is currently both patented and used in esophageal implants for cancer patients by the medical team. In the 2021–2022 academic year, the laboratory received a prototype of an esophageal prosthesis, which was the subject of a patent developed by a team of doctors from Colțea Hospital, registered in the Official Bulletin of Industrial Property OSIM of Romania under no. 130466/30.01.2017. At the same time, another result of the project team is represented by [15], which is a monitoring and control system with a data collection and statistics interface. Through the SIMBIO project [16], the project team brings closer innovative solutions for adaptive or stimulus-responsive functional elements, embeddable into hierarchical structures, which allow for beneficial structural modifications for design through silico techniques, which can be supported by artificial intelligence-assisted approaches.

As part of the project [16], algorithms were developed to analyze the influence of variations in prosthesis geometry and evaluate tissue response to the implant. Several relevant indicators were studied to understand the interaction between the prosthesis and the surrounding biological tissue. A predictive algorithm was also developed to detect anomalies in medical data sets, which allows for the automatic identification of deviations in the health status of patients, providing the possibility of early diagnosis and improving patient care. At the same time, strategies for multimodal treatment of esophageal cancer were explored, with the aim of optimizing therapeutic combinations and improving clinical outcomes. These strategies help the authors discover the integration of different treatment methods, such as surgery, chemotherapy, and radiotherapy, to increase their efficiency. The ultimate goal of the project [16] is to improve clinical outcomes by personalizing treatment according to the needs of each patient, based on information obtained from the developed algorithms and medical data analysis.

To continue the research, the authors proposed below two approaches through which controllers and data transmission can be used to be subsequently stored in databases and processed in statistical form.

The first is a configuration of hardware components composed of controllers and sensors, which can provide accuracy and precision in the esophageal prosthesis implant through data collection.

The second configuration of software and physical components composed of controllers and sensors is intended to collect data and indicate the level of inflammation and tolerance of the implant, which may reveal a possible relapse, which sends data to a CPSI.

The board used in the case study on both situations described is the Arduino Uno R3 ATMega328P-AU CH340G (Bitmi, Bucharest, Romania)—a versatile experimental platform widely used in scientific and technical research due to the integration of multiple inertial and environmental sensors. This board is, thus, a fundamental element in research and applied engineering projects, offering an optimal balance between performance, cost, and versatility.

### 3.1. Analysis of the Influence of Variations in Prosthesis Geometry

The analysis of the implant geometry may require the use of sensors capable of measuring dimensions, shapes, positions, and distances. Depending on the characteristics of the prosthesis, the following sensors are the most suitable:

- Ultrasonic sensor HC-SR04—The operating principle is based on ultrasonic waves to determine the distance to the skin (or, as the case may be, to another body/object). This sensor can be used to evaluate the dimensions and general shape of a body by successive measurements from different angles.
- GY-87 10DOF sensor (MPU6050, HMC5883L, BMP180)—It integrates an accelerometer, a gyroscope, a magnetometer, and a barometer, making it ideal for determining the kinetic and positional parameters of the prosthesis. Due to this, in the current application, it is proposed to be used for measuring tilt, orientation, and movement.
- Rotary encoder module KY-400—This sensor is intended for measuring rotation angles and rotational movements of the prosthesis. Due to its advantages, is useful in the analysis of moving bodies or in the study of rotation mechanisms.
- TCRT5000 IR Sensor Module—It uses the reflection of infrared light as its operating principle to detect the contours of an object placed on a flat surface. The sensor is used to identify and analyze shapes through the contrast between reflective and absorbent surfaces.
- KY-010 Light Interruption Sensor Module—Due to its physical properties, it can detect changes in light caused by the presence or absence of an object at a certain point. Strategically placed, this sensor is useful for identifying edges and delimiting the contours of the prosthesis.

**Figure 1:**
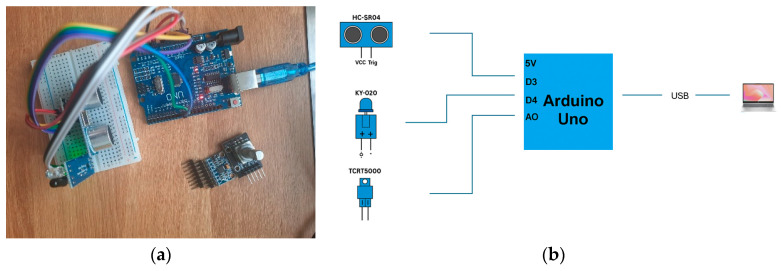
Prototype for analyzing the influence of variations in prosthesis geometry: (**a**) physical prototype; (**b**) circuit diagrams of the corresponding main electrical components.

The Figure 1 shows the prototype for analyzing the influence of variations in the geometry of the esophageal prosthesis, composed of the HC-SR04 ultrasonic sensor, KY-020 angle sensor, and TCRT5000 IR sensor.

The software code implemented for the three sensors on the work plate was designed to monitor and adjust the position of an intelligent esophageal prosthesis. The purpose of this prototype and its software implementation is to collect data on the positioning and stability of the implant in relation to the adjacent anatomical structures of the prosthesis and the patient. Below are presented the results of the software developed on the impact of each sensor on the geometry and functionality of the prosthesis.

The HC-SR04 sensor is used to measure the distance between the prosthesis and the walls of the esophagus. The data extracted in real time is processed, and it can be detected whether the prosthesis is in a stable position or if it has moved from the reference. By a sudden change in the distance, the software can indicate a migration of the prosthesis, which would require detailed medical intervention.

By using the KY-020 sensor, which detects changes in inclination, the alignment of the prosthesis can be assessed. This creates several cases that can be analyzed by either the software or the surgeon: if the prosthesis tilts excessively in one direction, this may indicate uneven pressure on the esophageal mucosa, which could lead to injury or discomfort for the patient. The software can send alerts if the position of the prosthesis deviates from the standard reference values, indicating the need for correction through an automatic adjustment mechanism or through a medical procedure.

In the prototype, the IR sensor (TCRT5000) is used to detect the presence of bodies that may interfere with the functionality of the prosthesis. By analyzing the data collected during the implant, an adjustment of the prosthesis position can be interpreted in such a way as to facilitate the passage of food and, thus, optimize the swallowing process.

### 3.2. Tolerance Feedback Prototype

Integrating sensors into such medical devices can significantly improve their monitoring and adaptability, allowing early detection of complications, optimization of positioning, and adjustment of parameters in real time. The most common sensors can be the following:

1. Sensors for detecting inflammation;
  - DS18B20—Accurate digital temperature sensor;
  - KY-013—Temperature sensor with NTC thermistor;
  - KY-028 (LM393)—Digital temperature sensor with comparator.
2. Sensors for analyzing body reactions (inflammation, stress, and sweating);
  - DHT11 (KY-015)—Temperature and humidity sensor;
  - Soil moisture sensor—adaptable for measuring skin perspiration.
3. Sensors for biomechanical analysis (inflammation, muscle stiffness, and tremor):
  - SW-18015P—Vibration sensor;
  - KY-031—Vibration sensor module;
  - GY-87 (MPU6050)—Accelerometer and gyroscope (detects movements and joint stiffness).

**Figure 2:**
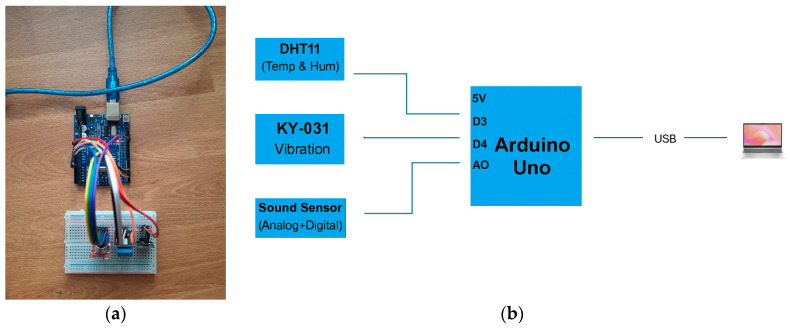
Prototype for early detection of complications: (**a**) physical prototype; (**b**) circuit diagrams of the corresponding main electrical components.

Figure 2 shows the prototype for the tolerance analysis application and early detection of complications. In the developed code, the additional library required for the operation of the sensors is DHT.h. This is used and introduced for communication with the DHT11 temperature and humidity sensor; being indispensable for compiling the code, it provides functions for reading temperature and humidity in a simplified way. The library can be installed from the Arduino Library Manager or downloaded from official sources, such as GitHub (https://docs.arduino.cc/libraries/dht-sensor-library/ (accessed on date 15 Jule 2025)). This research uses the DHT11 library downloaded from the Arduino Library Manager.

The use of the DHT11 sensor allows the recording of temperature and humidity in real time. These measurements are relevant for the detection of local inflammation, which may indicate an adverse reaction of the tissues to the implant. The increase in temperature around the prosthesis may be a sign of an inflammatory process, and changes in esophageal humidity may influence the adhesion of the prosthesis and the formation of pathological secretions, such as excessive mucus. These data are essential for the early diagnosis of infections or the adaptation of post-implantation treatment.

By integrating the KY-031 vibration sensors, the movement of the prosthesis and its interaction with the esophagus can be monitored. These sensors can detect abnormal oscillations of the implant caused by esophageal peristalsis or involuntary contractions. In the case of a positioned or unstable prosthesis, vibrations may indicate irritation of the esophageal mucosa, which could lead to tissue erosions or discomfort for the patient. Thus, by analyzing vibrations, the positioning and stability of the prosthesis can be improved, reducing the risk of subsequent complications.

The integration of DUFAFF_MIC_SOUND, a sound sensor (which sends data both digitally and analogically), allows the recording of the sound level generated by the internal movements of the esophagus. The main function that this sensor performs in the application proposed by the authors is to send data about the behavior of the prosthesis during the swallowing process. These measurements can provide information about the efficiency of swallowing, thus, detecting obstructions or gastroesophageal reflux.

By analyzing the data distribution graph, an abnormal sound level, or sudden fluctuations can be identified that could signal esophageal dysfunction or incomplete adaptation of the prosthesis to the patient’s anatomy.

### 3.3. Interface in Cyber-Physical System Implementation

A typical CPSI could integrate sensors to measure temperature, humidity, vibration, movement, and other physiological signals within the esophageal prosthesis. This would allow for constant monitoring of the patient’s condition and the interaction of the prosthesis with the esophageal environment, identifying potential complications or problems (inflammation, adverse reactions, prosthesis instability).

CPSI in the field of medicine, and especially in esophageal cancer is a concept quite far from current studies. Few works address the field of CPSI due to several medical factors such as difficult-to-access anatomy (deep location of the organ), high variability between patients, and the need for precise imaging diagnosis combined with biopsies. But in addition to these factors, it can be mentioned that at the current level a standard for safety and extremely strict regulations are not indicated. As small steps in this direction, the authors of the work [17] designed a photoelectrochemical biosensor (PEC), without supply voltage, based on the Cu_2_O/PEDOT:PSS/ZnO nanocomposite. The goal was to detect esophageal cancer cells and classify them based on the level of aggressiveness.

With this discovery, we can adapt the idea of CPSI from this article with future advances in biosensors that can be implanted in the esophageal area.

A group of researchers analyzed the current role of artificial intelligence in the management of esophageal cancer [18] and focused on one of the future perspectives of obtaining wearable devices, simultaneously measuring self-reported adverse events, emergency department visits, and chemotherapy completion rates through an application to ultimately develop an artificial intelligence-based complication prediction algorithm based exclusively on biomarkers (NCT05937477).

Thus, in this sense, an interface that can be used by both the patient (local) and the physician (remote) is proposed below.

**Figure 3:**
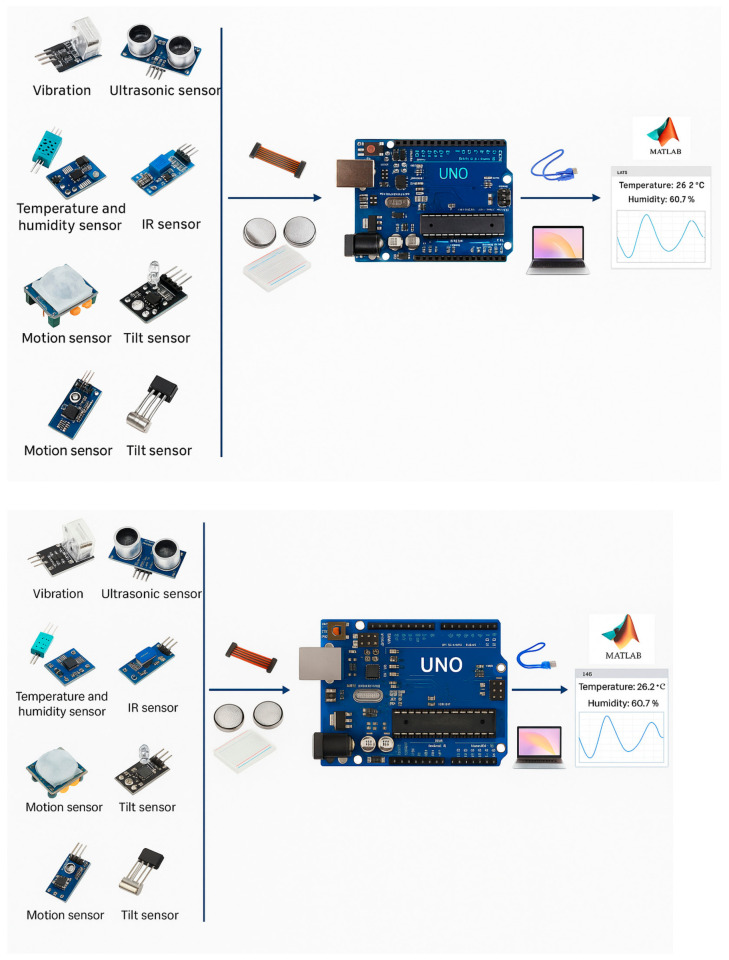
SIMBIO system architecture.

Figure 3 represents a functional block diagram of the Cyber-Physical System Interface (CPSI) used for sensor integration and data analysis and illustrates, from left to right the following:

1. Sensors (Input Layer): Vibration sensor, ultrasonic sensor, temperature and humidity sensor, infrared (ir) sensor, motion sensor, tilt sensor—these modules form the sensory subsystem for detecting environmental and physiological parameters relevant to prosthesis behavior and tissue response;
2. Signal conditioning and transmission (interface layer): breadboard and jumper wires—ensure flexible prototyping and interconnection between sensors and the controller, enabling real-time signal routing and data acquisition;
3. Processing unit (control layer): Arduino Uno microcontroller board—acts as the central processing unit (CPU), collecting raw sensor data and performing initial filtering, analog-to-digital conversion, and communication handling;
4. Communication and visualization (output layer): USB interface to laptop—facilitates serial data transmission from the Arduino to the host system. MATLAB GUI interface—used for real-time visualization, data logging, and analysis, providing a scientific environment for evaluating implant geometry, motion, and surrounding tissue response.

Within the SIMBIO project, the first version of CPSI in vivo was developed, which will have improvements after a detailed analysis together with complex teams and feedback from stakeholders. The interface at this time in the current version, at the graphics level, was created with Design App in MATLAB, version R2021b. Desing App was chosen for the graphical interface of the application due to several advantages it offers in the context of the development of an esophageal prosthesis system:

1. It allows the fast and efficient creation of graphical interfaces (GUI—Graphical User Interface) through a drag-and-drop approach;
2. It has direct interaction with data and simulation models. Within an esophageal prosthesis system, this means that data collected from sensors can be processed and visualized instantly, and users can observe the evolution of parameters, such as temperature, vibrations, or humidity, in an interactive and visual way;
3. Allows customization of the interface appearance, providing flexibility to meet the specific needs of a medical system;
4. Allows integrating advanced functionalities, such as data processing algorithms, statistical analysis, or machine learning, directly into the graphical application.

The system is organized so that each sensor is connected to the microcontroller through dedicated pins. The hardware configuration is achieved by assigning the trigPin and echoPin pins for the ultrasonic sensor, tiltPin for the KY-020 sensor, and irPin for the IR sensor. Distance measurement using the ultrasonic sensor is achieved by generating a short pulse on the sending pin (trigPin), followed by measuring the time required for the echo to return to the receiving pin (echoPin). Within the software implementation, communication between the microcontroller and an external system (PC or other monitoring device) is achieved through a UART (Universal Asynchronous Receiver-Transmitter) serial interface. To ensure efficient data transmission, the Serial.begin (9600); function initializes the baud rate to 9600 bps (bits per second), a value chosen to maintain a balance between transfer speed and communication stability. This value is compatible with most microcontrollers and avoids synchronization errors or data loss, which can occur at higher rates without appropriate hardware.

During project implementation, the microcontroller is connected to a computer via a USB serial port, which is mapped as COM5 on the host system.

Serial communication between Arduino and MATLAB is an efficient method to transfer sensor data in a format that can be analyzed and processed for optimizing the design of the esophageal prosthesis and for continuous monitoring of the patient’s condition. This approach combines hardware with software to create an intelligent and adaptable system that improves diagnostic accuracy and treatment efficiency. Some of the functions in the Arduino code were essential for transmitting data to a computer or application that processes and analyzes this information and realizes CPSI. Specifically, these functions are used to send measured data from sensors (such as temperature, vibration, or humidity) via the serial port to software on the computer (e.g., MATLAB or a custom application).

To visualize and monitor real-time sensor data, a custom graphical interface was developed using MATLAB App Designer. This tool enables the creation of interactive applications with graphical elements (widgets), including plots, buttons, and labels. The GUI (Graphical User Interface) contains a Push Button, which is associated with a callback function. When the user clicks this button, the application performs the following:

- establishes a serial connection with the Arduino board via COM5;
- reads sensor data in real-time from the microcontroller;
- parses the serial input and extracts the individual sensor values;
- stores the data in vectors dedicated to each sensor type;
- dynamically updates the graph, reflecting the current sensor readings over the selected time interval.

**Figure 4:**
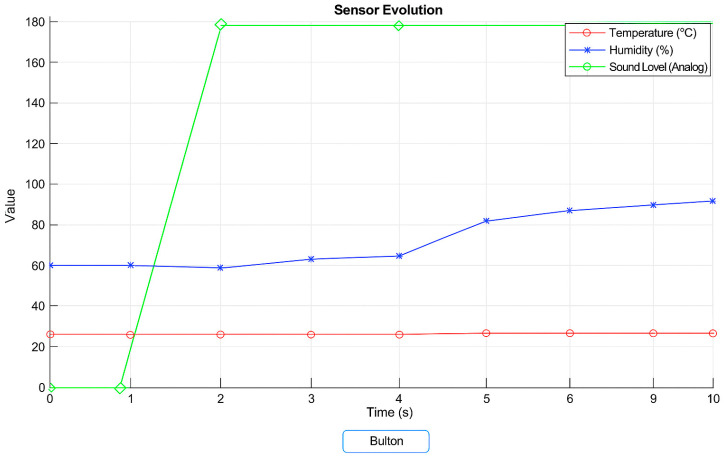
Interface in Cyber-Physical System graphic representation.

Although the same sensors described in Section 3.2 are used in this implementation, Figure 4 presents them using simplified, user-friendly terminology. This choice was made intentionally, as the interface illustrated in Figure 4 is designed for non-technical users such as medical staff or patients. The original product codes and detailed sensor specifications remain listed in Section 3.2 to maintain technical accuracy, while the interface focuses on usability and intuitive understanding.

Figure 4 shows the data from three sensors (temperature, humidity, and swallowing sound). Also in the image is a button, which by pressing it, this button associates with a callback that triggers this complete process. By pressing the button, the MATLAB application will open the connection with Arduino, read the data from the sensors connected to the microcontroller, and continuously update the graph in the interface as new values are received. Initialization of the vectors for storing data that will store the measured data for each sensor over 10 s (time chosen for testing, but a relevant time for the application can be chosen hours/days, etc.). As time passes (in this case, for 10 s), the application reads a line of data from Arduino. The data is then processed to extract the numerical values corresponding to each sensor. After reading each set of data, the graph is updated to reflect the evolution of the sensors over time.

The MATLAB code for this application is implemented in the App Designer environment and includes the GUI design and the backend code for serial communication and data visualization.

## 4. Conclusions

The presented esophageal prosthesis can become an intelligent and adaptable device, capable of providing real-time feedback on the interaction with the medical environment. This approach contributes to the early diagnosis of complications, the optimization of the prosthesis positioning, and the improvement of the patient’s quality of life.

The presented interface can be easily adapted to handle more complex visualizations and advanced algorithms, such as regression analysis, machine learning models, or other data-driven techniques. Using the robust computational and graphics capabilities of MATLAB, the system can process and display complex data sets in real time, providing valuable information about the behavior and performance of external devices, such as a prosthetic implant. By integrating more sophisticated algorithms, the interface can be extended to provide predictive analytics, trend analysis, or even optimization tasks tailored to specific biomedical applications.

Currently, there are no clearly defined clinical guidelines, regulations, or certification standards for the use of wearable or implantable devices with integrated sensors in the postoperative monitoring of esophageal cancer. This lack of regulation represents a major obstacle to the scalability and transposition into clinical practice of technological solutions such as CPSI. For this reason, it is essential for such initiatives to be approached through a solid interdisciplinary collaboration, with a team formed by engineers, physicians, surgeons, biomedical device researchers, and representatives of implantable medical technology companies. Only through joint effort can safe, standardized, and effective solutions be developed to meet the complex needs of patients and therapeutic teams for esophageal diseases.

Future works will integrate an interface enabling machine learning algorithms to predict future prosthesis abnormalities or recurrence data and also analyze the feedback of sensors during interventions on the prosthesis. The use of biomedical-grade sensors will be considered, as well as the construction of artificial muscles to create a real monitoring, control, and actuation system.

## Data Availability

No new data were created or analyzed in this study. Data sharing is not applicable to this article.

## References

[1] Wang Y., Mukkamalla S.K.R., Singh R., Lyons S. (2024). Esophageal Cancer. StatPearls.

[2] Denecke C., Pratschke J., Raakow J. (2022). Current Issues and Future Technologies in Esophageal Cancer Surgery. J. Clin. Med..

[3] Jung K., Haug R.M., Wang A.Y. (2024). Advanced Esophageal Endoscopy. Gastroenterol Clin. N. Am..

[4] Goda K., Abe K., Kanamori A., Kondo M., Kojimahara S., Kanazawa M., Tanaka T., Nagashima K., Suzuki T., Yamamiya A. (2022). Advanced Endoscopy for Benign Esophageal Disease: A Review Focused on Non-Erosive Reflux Disease and Eosinophilic Esophagitis. Healthcare.

[5] Spadaccini M., Vespa E., Chandrasekar V.T., Desai M., Patel H.K., Maselli R., Fugazza A., Carrara S., Anderloni A., Franchellucci G. (2022). Advanced imaging and artificial intelligence for Barrett’s esophagus: What we should and soon will do. World J. Gastroenterol..

[6] Deboever N., Jones C.M., Yamashita K., Ajani J.A., Hofstetter W.L. (2024). Advances in diagnosis and management of cancer of the esophagus. BMJ.

[7] Wykypiel H., Gehwolf P., Kienzl-Wagner K., Wagner V., Puecher A., Schmid T., Cakar-Beck F., Schäfer A. (2024). Clinical implementation of minimally invasive esophagectomy. BMC Surg..

[8] Bjelovic M., Gunjic D., Babic T., Veselinovic M., Djukanovic M., Potkonjak D., Milosavljevic V. (2024). Safe Transition from Open to Total Minimally Invasive Esophagectomy for Cancer Utilizing Process Management Methodology. J. Clin. Med..

[9] Reddy A.T., Lee J.P., Leiman D.A. (2024). Measuring and improving quality in esophageal care and swallowing disorders. Dis. Esophagus..

[10] Qiu L.H., Liang S.H., Wu L., Huang Y.Y., Yang T.Z., Li C.Z., Huang X.L., Zhong J.D., Ma G.W. (2024). Longitudinal assessment of quality of life indicators and prognosis in esophageal cancer patients with curative resection. J. Thorac. Dis..

[11] Pannala R., Krishnan K., Watson R.R., Vela M.F., Abu Dayyeh B.K., Bhatt A., Bhutani M.S., Bucobo J.C., Chandrasekhara V., Copland A.P. (2022). Devices for esophageal function testing. Gastrointest. Endosc..

[12] Butt I., Kasmin F. (2023). Esophageal pH Monitoring. StatPearls.

[13] Mîndra T., Anghel A.M. Hybrid Co-Simulation Framework for Cyber-Physical Systems-Based Applications. Proceedings of the 2024 International Conference on Applied Mathematics & Computer Science (ICAMCS).

[14] Mîndra T., Anghel A.M. (2024). Cyber–Physical Perception Interface for Co-Simulation Applications. Sensors.

[15] Energy Efficiency and Increased Comfort within the Residential Home for the Elderly in Alba Iulia, EfiGeo4AI, EEA and Norway Grants 2014–2021. https://www.sis.ro/efigeo4ai/.

[16] SIMBIO. https://upb.ro/wp-content/uploads/2022/11/Lista-proiecte-finantate-dupa-suplimentarea-bugetului.pdf.

[17] Tseng K.W., Hsiao Y.P., Jen C.P., Chang T.S., Wang H.C. (2020). Cu_2_O/PEDOT:PSS/ZnO Nanocomposite Material Biosensor for Esophageal Cancer Detection. Sensors.

[18] Mela E., Tsapralis D., Papaconstantinou D., Sakarellos P., Vergadis C., Klontzas M.E., Rouvelas I., Tzortzakakis A., Schizas D. (2025). Current Role of Artificial Intelligence in the Management of Esophageal Cancer. J. Clin. Med..

